# The NLRP3 inflammasome: contributions to inflammation-related diseases

**DOI:** 10.1186/s11658-023-00462-9

**Published:** 2023-06-27

**Authors:** Ying Chen, Xingyan Ye, Germaine Escames, Wangrui Lei, Xin Zhang, Meng Li, Tong Jing, Yu Yao, Zhenye Qiu, Zheng Wang, Darío Acuña-Castroviejo, Yang Yang

**Affiliations:** 1 Department of Hematology, The First Affiliated Hospital of Xi’an Jiaotong University, Xi’an, China; 2 Key Laboratory of Resource Biology and Biotechnology in Western China, Ministry of Education, Faculty of Life Sciences and Medicine, Northwest University, Xi’an, China; 3 Department of Neurology, Xi’an No. 3 Hospital, The Affiliated Hospital of Northwest University, Xi’an, China; 4 Biomedical Research Center, Health Sciences Technology Park, University of Granada, Avda. del Conocimiento s/n, Granada, Spain; 5 Ibs. Granada and CIBERfes, Granada, Spain; 6 UGC of Clinical Laboratories, University San Cecilio’s Hospital, Granada, Spain; 7 Department of Cardiology, Affiliated Hospital, Yan’an University, Yan’an, China; 8 Department of Cardiothoracic Surgery, Central Theater Command General Hospital of Chinese People’s Liberation Army, Wuhan, China

**Keywords:** NLRP3 inflammasome, Innate immunity, Inflammatory disease

## Abstract

The NOD-like receptor protein 3 (NLRP3) inflammasome is a protein complex that regulates innate immune responses by activating caspase-1 and the inflammatory cytokines interleukin (IL)-1β and IL-18. Multiple studies have demonstrated the importance of the NLRP3 inflammasome in the development of immune and inflammation-related diseases, including arthritis, Alzheimer’s disease, inflammatory bowel disease, and other autoimmune and autoinflammatory diseases. This review first explains the activation and regulatory mechanism of the NLRP3 inflammasome. Secondly, we focus on the role of the NLRP3 inflammasome in various inflammation-related diseases. Finally, we look forward to new methods for targeting the NLRP3 inflammasome to treat inflammation-related diseases, and provide new ideas for clinical treatment.

## Introduction

The innate immune system acts as the first line of host defense to trigger the adaptive immune response. This system initiates downstream inflammatory cascades in response to noxious stimuli through germline-encoded pattern recognition receptors (PRRs). PRRs are distributed in the cell membrane and cytoplasm, playing a prominent role in initiating innate and adaptive immunity. Their main function is to produce pro-inflammatory cytokines and interferons by transcription [1, 2]. The activating factors of PRRs include pathogen-associated molecular patterns (PAMPs) and damage-associated molecular patterns (DAMPs), which are endogenous molecules derived from dying cells [3]. However, inappropriate activation of PRRs causes long-term inflammation and even leads to autoimmune and inflammatory diseases [4, 5]. As an important PRR in the cytoplasm, the NOD-like receptors (NLR) family is instrumental in the inflammatory response and has attracted wide attention in recent years.

Based on the nature of nitrogen-terminal domains, members of the NLRs family are subdivided into four subfamilies, including NLRA, NLRB, NLRC, and NLRP. NLRs family activates multiple downstream signals, promoting inflammasome assembly and inflammatory response [6]. In recent years, the NOD-like receptor protein 3 (NLRP3) and the inflammasome assembled from it is the most focused on inflammasome. However, the current understanding of the NLRP3 inflammasome still has unresolved questions, such as its structure and activation mechanism. In addition, increasing evidence suggests that activation of the NLRP3 inflammasome is involved in the pathological process of various inflammatory diseases [7–9]. The activation of the NLRP3 inflammasome aggravates oxidative stress and vascular endothelial dysfunction, and accelerates the pathological process of cardiovascular diseases [10]. In rheumatoid arthritis, the deletion of NLRP3 and its downstream components significantly reduces inflammation and cartilage destruction [11]. Furthermore, in the microglia of the nervous system, the NLRP3 inflammasome can sense protein misfolding deposition or amyloid β (Aβ) aggregation and be activated to promote the occurrence and progression of neurodegenerative diseases [12]. The important role of the NLRP3 inflammasome in human diseases further indicates that it is of great clinical significance to study the mechanism of its involvement in diseases and targeted drug therapy.

In this review, we first describe the composition of the NLRP3 inflammasome and elaborate on the activation mechanism of the NLRP3 inflammasome, including both canonical and non-canonical activation pathways. In addition, we highlight the pivotal role that the NLRP3 inflammasome plays in inflammation-related diseases. Finally, we look to the future of the NLRP3 inflammasome as a promising therapeutic target for disease.

## NLRP3 inflammasome

The inflammasome is a crucial component of the innate immune system’s response to pathogens, which consists of a set of cytoplasmic multiprotein complexes. Inflammasomes activate specific caspase proteases in response to infection or noxious stimuli [13]. A variety of inflammasomes, including NLRP1, NLRP3, NLRC4, and AIM2, have been identified [6, 14]. Different inflammasomes have distinct stimulatory signals but have very conserved downstream effects, especially in the activation of caspase-1, which in turn triggers the three key substances: pro-interleukin-1β (IL-1β), pro-IL-18, and gasdermin D (GSDMD) [13, 15]. Most inflammasomes are activated only by one or a few highly specific agonists (e.g., AIM2 is activated only by DNA from DNA viruses or bacteria), whereas NLRP3 can respond to a variety of agonists that are abundant in source and unrelated in structure and chemical properties. As such, it possesses the broadest functional scope of all inflammasomes in both innate and adaptive immune systems [16, 17]. The NLRP3 protein belongs to the family of nucleotide-binding oligomerization domain-like receptors (NLRs) [18]. The NLRP3 protein contains a leucine-rich repeat (LRR) domain at the carboxyl terminus, a pyrin domain (PYD) at the amino terminus, and a nucleotide-binding domain (NACTH) in the center domain [18].

The inflammasome fails to assemble successfully without immune activators, which is due to an internal interaction between the NACHT domain and LRRs replacing the interaction between NLRP3 and the apoptosis-associated speck-like protein (ASC) [19]. However, when immune activators, such as PAMPs, DAMPs, and various exogenous or environmental stimuli are present, the internal structure of NLRP3 opens up, allowing PYD–PYD interaction between NLRP3 and ASC. Subsequently, the caspase recruitment domain (CARD) of ASC binds to the CARD on pro-caspase-1 to form the NLRP3 inflammasome. The inflammasome of NLRP3 produce mature caspase-1 that cleaves pro-IL-1β and pro-IL-18 to produce corresponding mature cytokines [20, 21].

## Activation mechanism of the NLRP3 inflammasome

### Canonical activation of NLRP3

**Fig. 1:**
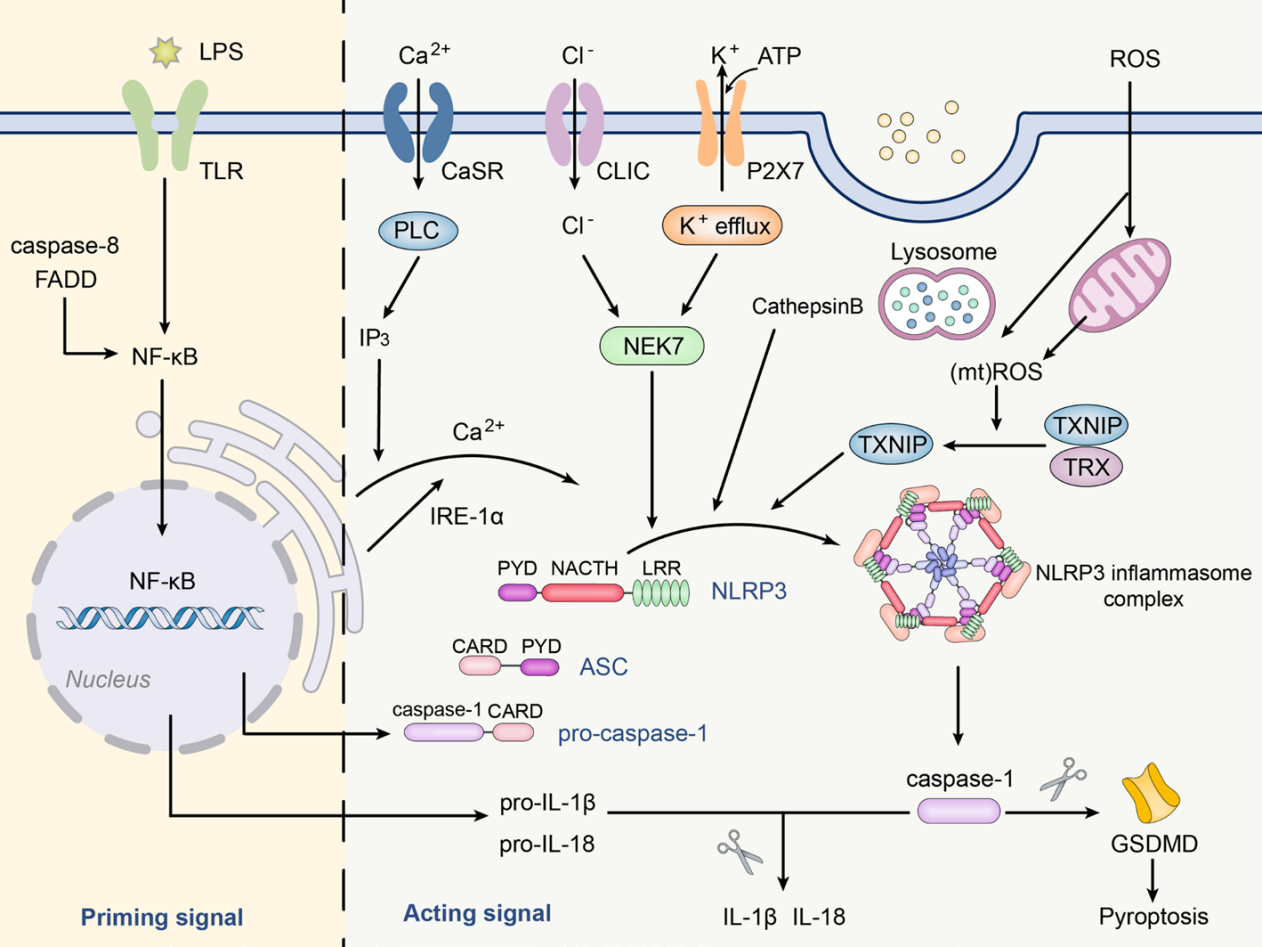
Canonical activation of the NLRP3 inflammasome. The NLRP3 inflammasome comprises NLRP3 protein, ASC, and pro-caspase-1. The activation of the NLRP3 inflammasome includes both canonical and noncanonical methods. Multiple mechanisms activate the inflammasome, such as P2X7 channel-mediated K^+^ efflux, mitochondrial dysfunction, mitochondria active oxygen species (mtROS) release, lysosomal disruption, and subsequent cathepsin B release

The canonical activation of NLRP3 inflammasome is a two-step process consisting of priming and activation signals (Fig. 1).

#### Step 1: priming signal

Priming signals recognize DAMPs or PAMPs though Toll-like receptors and activate the NF-κB signaling pathway, upregulating IL-1β and NLRP3 protein expression. During priming, apoptosis-related signaling molecules caspase-8 and Fas-associated with death domain protein (FADD) act as regulators of NLRP3 inflammasome upstream signaling, affecting transcriptional initiation and post-translational activation of NLRP3 inflammasome pathways, independent of its apoptotic function [22]. Caspase-8 interacts with the inhibitory kappa B kinase (IKK) complex. The IKK complex is a fundamental component of activating the NF-κB pathway and promotes NF-κB transcription and translocation in initiation [23]. The priming signal has at least two functions for NLRP3 inflammasome activation. Firstly, up-regulated expression of related components, including NLRP3 protein, pro-caspase-1, and pro-IL-1β [24–26], and secondly to induce multiple posttranslational modifications (PTMs) of NLRP3, including ubiquitination, SUMOylation, and phosphorylation [27–29].

#### Step 2: activating signal

Various molecular or cellular events are important causes of NLRP3 inflammasome activation, including lipopolysaccharide (LPS), extracellular ATP, crystallization, ion flux, lysosomal damage, and production of reactive oxygen species (ROS) induced by mitochondrial dysfunction. Once activated, the inflammasome initiates a series of downstream reactions [30].

### K^+^ efflux

Early studies have shown that K^+^ efflux is a common phenomenon that activates the NLRP3 inflammasome. NLRP3 agonists first trigger cell membrane permeation of K^+^ and Na^+^, which in turn activates the NLRP3 inflammasome. Notably, a low level of intracellular K^+^ concentration is sufficient to activate the NLRP3 inflammasome, whereas an increase in intracellular Na^+^ concentration is involved in regulation but is not necessary for NLRP3 inflammasome activation [31]. K^+^ efflux mediated by P2X7 receptor channels is an efficient pathway to activate the NLRP3 inflammasome [32]. The P2X7 receptor channel is a plasma membrane channel directly activated by extracellular ATP. ATP binds to the P2X7 receptor to form a DAMP and then opens the channel to cause K^+^ transmembrane efflux, ultimately activating the NLRP3 inflammasome. Similarly, in addition to the P2X7 receptor pathway, some two-pore domain potassium (K2P) channels can also cause the NLRP3 inflammasome activation in a K^+^ efflux-dependent manner. Fifteen K2P channel genes have been identified in the human genome, and six distinct subfamilies can be distinguished based on their structural and functional characteristics, including TASK, TALK, TRESK, TREK, THIK, and TWIK [33]. Among them, the ATP-induced two-pore domain weak inwardly rectifying K^+^ channel 2 (TWIK2) causes K^+^ efflux, leading to NLRP3 inflammasome activation in macrophages. It should be noted that there is a synergic effect between P2X7 and TWIK2, the former changes the membrane potential through Ca^2+^ and Na^+^ influx, while the latter activates the NLRP3 inflammasome through K^+^ efflux [34]. THIK-1, another member of the K2P channel family, is also involved in regulating NLRP3 inflammasome activation, and this process occurs downstream of the ATP/P2X7 receptor channel [35].

Furthermore, the mechanism of K^+^ efflux-mediated activation of the NLRP3 inflammasome involves the binding of never in mitosis gene A-related kinase 7 (NEK7) protein and NLRP3. NEK7 forms a molecular complex with NLRP3 in the cytoplasm, and its catalytic domain binds to the NLRP3 protein [36]. At present, the exact mechanism of K^+^ activation of the NLRP3 inflammasome is not completely clear, and no studies have shown that K^+^ directly interacts with the NLRP3 protein. Instead, it is believed that the NEK7–NLRP3 interaction may cause structural changes in the NLRP3 protein, resulting in K^+^-induced NLRP3 inflammasome activation [37]. To sum up, further exploration is required to fully understand the role of K^+^ efflux in activating the NLRP3 inflammasome.

### Mitochondrial dysfunction and production of ROS

Mitochondrial dysfunction is also a key contributing factor for the NLRP3 inflammasome activation. The signals released by mitochondrial damage mainly include mitochondrial DNA (mtDNA) and mitochondria active oxygen species (mtROS). NLRP3 protein functions as a sensitive sensor, triggering downstream caspase-1 activation and releasing the cytokine IL-1β in response to cellular danger signals associated with mitochondrial damage [38, 39].

Shimada et al. first proposed that oxidized mitochondrial DNA (OX-mtDNA) could induce the activation of the NLRP3 inflammasome during apoptosis. In the presence of NF-κB, ATP induces mitochondrial dysfunction and cell apoptosis, leading to the release of OX-mtDNA from mitochondria. In the cytoplasm, OX-mtDNA binds and activates the NLRP3 inflammasome [40]. One study found that cytidine monophosphate kinase 2 (CMPK2) is involved in cytoplasmic OX-mtDNA-induced NLRP3 inflammasome activation. CMPK2 transcription provides deoxyribonucleotides for mtDNA synthesis, which lays the foundation for NLRP3 agonists to stimulate OX-mtDNA production [41]. Xue et al. demonstrated that increased levels of OX-mtDNA and aggravated mitophagy activate the NLRP3 inflammasome in rat liver [42]. The above results indicated that OX-mtDNA in the cytoplasm can successfully activate NLRP3 inflammasome.

In addition, mtROS not only directly regulates the assembly process of inflammasome, but also indirectly regulates the activity of inflammasome by affecting cytoplasmic proteins [43]. Specifically, mtROS mediates the dissociation of thioredoxin-interacting protein (TXNIP) from thioredoxin and then interacts with NLRP3 protein to activate the NLRP3 inflammasome [44]. The activation of the NF-κB pathway by ROS not only mediates the assembly of the NLRP3 inflammasome but also directly promotes the expression of tumor necrosis factor-α (TNF-α), pro-IL-1β, IL-6, and other inflammatory factors [45]. Another study showed that mitochondrial dysfunction activates the NLRP3 inflammasome through the synthesis of phosphatidylglycerol, but not ROS production. Phosphatidylglycerol directly binds to NLRP3 and interfering with its synthesis can specifically inhibit the activation of the NLRP3 inflammasome [46]. Furthermore, a recent study suggested that other organelles rather than mitochondria influence the activation of the NLRP3 inflammasome. After being stimulated, no morphological changes of NLRP3 puncta or colocalization of mitochondria with NLRP3 puncta are observed in mitochondria, especially NLRP3 puncta localized on the trans-golgi network (TGN). The recruitment of NLRP3 protein by the dispersive TGN as a scaffold leads to the polymerization of the adapter protein ASC, to activate downstream cascades [47]. The specific mechanisms of the NLRP3 inflammasome activation by mitochondrial dysfunction and resulting ROS production need to be further studied.

### Lysosome rupture

An earlier study finds that lysosomes can uptake silica and aluminum salt in the alveoli, causing their swelling and rupture with concomitant exudation of the contents. This chain of events subsequently triggers activation of the NLRP3 inflammasome [48].

CD36 is a pattern recognition receptor that mediates the endocytosis of soluble ligands. CD36 are transformed into crystals or fibrils within cells, leading to lysosomal destruction and then the activation of NLRP3 inflammasome activates [49]. In a mouse model of Alzheimer’s disease (AD), the fibrillar peptide amyloid beta (Aβ) can be phagocytosed by lysosomes leading to lysosomal damage and cathepsin B release, which in turn activates the NLRP3 inflammasome [50]. Cathepsin B release not only directly activates the NLRP3 inflammasome but also mediates mitochondrial dysfunction, resulting in mtROS-triggered NLRP3 inflammasome activation [51]. Similarly, in a study of a mouse model of liver fibrosis, LPS/ATP treatment increases the contents of cathepsin B and ROS and activates NLRP3 inflammasome, while LPS/ATP and cathepsin B inhibitors fails to increase levels of NLRP3 and IL-1β [52].

### Other activation signals

In addition to the three activation signals that have been previously described, there are several other signals that impact the initiation and activation of the NLRP3 inflammasome. For example, irrecoverable endoplasmic reticulum stress reduces the expression of microRNA 17 (miR17) through over-activated inositol-requiring enzyme 1. The reduction of miR17 leads to the stable expression of TXNIP protein, which activates the NLRP3 inflammasome and exacerbates apoptosis[53]. In addition to K^+^, other ion streams (including Na^+^, Ca^2+^, Mg^2+^, Mn^2+^, Zn^2+^, Fe^2+^, and Cl^−^) also participate in regulating the activation of NLRP3 inflammasome in macrophages [54].

### Noncanonical activation of NLRP3 inflammasome

**Fig. 2:**
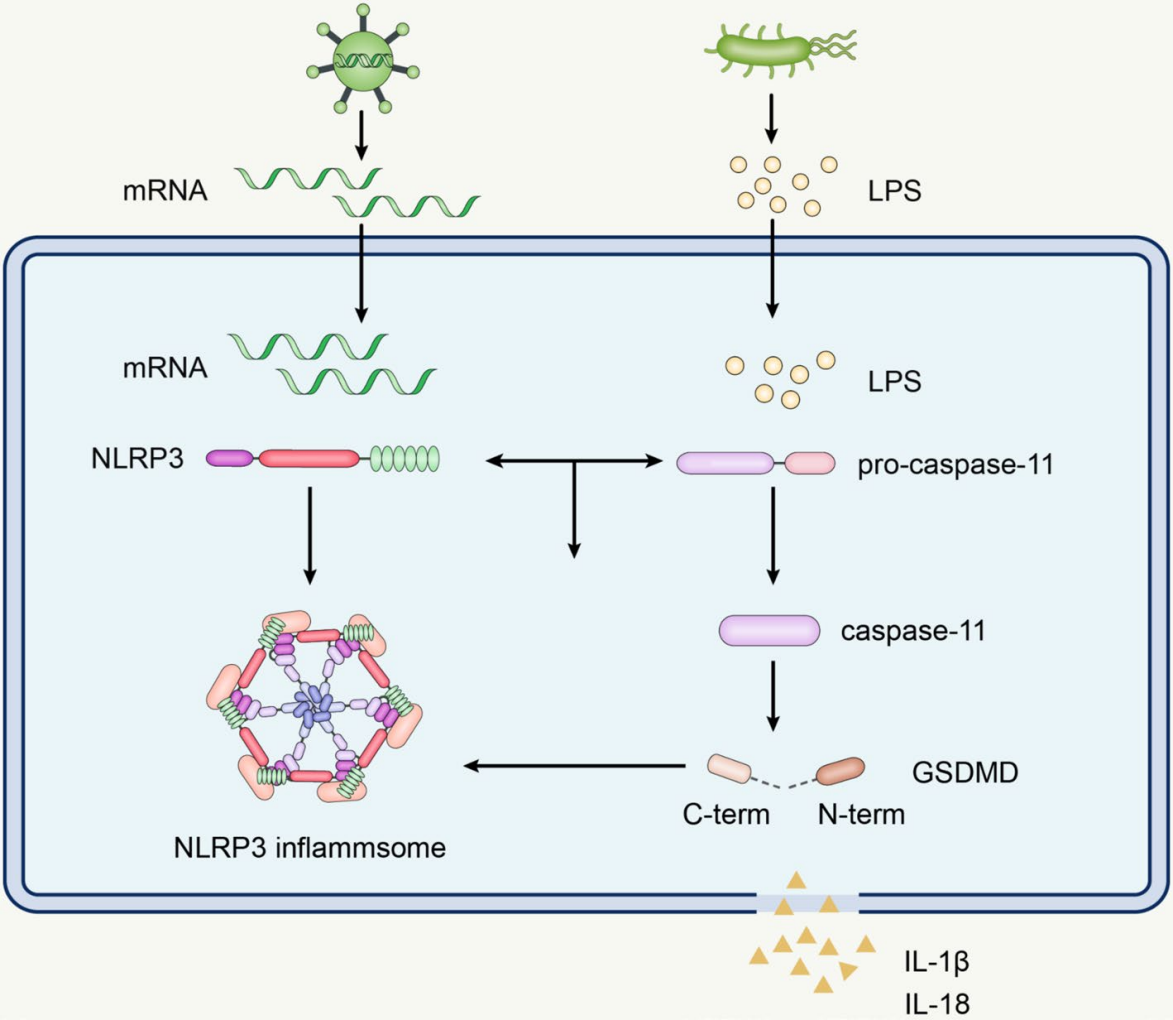
Noncanonical activation of the NLRP3 inflammasome. LPS can induce noncanonical activation of the NLRP3 inflammasome. LPS enters the cytoplasm by endocytosis or transfection, and then binds and activates pro-caspase-11, which subsequently cleaves GSDMD to promote pyroptosis, and activates the NLRP3 inflammasome

The noncanonical activation of the NLRP3 inflammasome is mainly mediated by lipopolysaccharide (LPS) on the surface of Gram-negative bacteria. Direct entry of LPS into the host cell cytoplasm occurs via endocytosis or transfection, without the requirement of Toll-like receptor 4 (TLR4) [55]. LPS binds and activates pro-caspase-11 upon entry into the cell, which further induces GSDMD cleavage. GSDMD belongs to the gasdermin family and has a functional N-terminal fragment and a self-inhibitory C-terminal fragment. Intracellular LPS binds and activates pro-caspase-11, this further induces GSDMD cleavage. The amino-terminal GSDMD fragment leads to pyroptosis and activates the NLRP3 inflammasome [56, 57]. Pyroptosis is a proinflammatory programmed cell death that results in the release of cellular contents. It is noteworthy that the noncanonical activation of the NLRP3 inflammasome in macrophages requires the cooperation of LPS and mRNA from Gram-negative bacteria. NLRP3 detects bacterial mRNA while LPS binds to pro-caspase-11. This interaction between pro-caspase-11 and NLRP3 enhances noncanonical activation of the NLRP3 inflammasome [58] (Fig. 2).

The activation of noncanonical NLRP3 inflammasome is dependent on GSDMD. The activation of GSDMD may not be potent enough to promote cell death, but it is robust enough to cause K^+^ efflux and activate NLRP3 inflammasome [59]. GSDMD not only alters the permeability of the cell membrane, but also creates pores in the mitochondrial membrane and causes the release of mtDNA. The presence of mtDNA and LPS allows Nur77 to bind to NLRP3 and activate NLRP3 inflammasome [60]. Additionally, once activated, the NLRP3 inflammasome promotes the cleavage of GSDMD by caspase-1 to produce N-GSDMD, which drives the onset of pyroptosis [61]. Pyroptosis caused by GSDMD leads to the release of IL-1β, promoting local inflammation [62]. GSDMD and NLRP3 reinforce each other, creating an amplified signaling loop. Precisely modulating this loop will provide new avenues for treating diseases.

## The role of the NLRP3 inflammasome in inflammation-related diseases

NLRP3 inflammasome is widely involved in the pathological development of a variety of inflammation-related diseases. In this section, related diseases in several human vital organs/tissues are discussed (Table 1).

**Table 1:** The role of NLRP3 inflammasome in diseases

Disease	Model/patients	Mechanism	References
CAPS	Patients	Gain-of-function mutations of the *NLRP3* gene result in the excessive activation of NLRP3 inflammasome that causes sustained and uncontrolled release of IL-1β.	[64]
CAPS	Patients and crossed *Nlrp3*^A350V^*NeoR*^*fl/*+^ mice with neutrophil-specific *MRP8-Cre* mice	NLRP3 inflammasome activation-induced cytokine IL-1β specifically enhances in neutrophils.	[68]
FMF	Patients	The monocytes from FMF patients secrete more NLRP3-dependent IL-1β.	[76]
AD	THY-Tau22 transgenic mice crossed with either Pycard-knockout mice (named *Asc*^−/−^) or Cias1-knockout mice (named *Nlrp3*^−/−^)	NLRP3 inflammasome activation induces tau hyperphosphorylation and aggregation.	[86]
PD	Patients	NLRP3 inflammasome activation aggravates the clinical features of PD through neuroinflammation.	[95]
PD	MPTP-induced PD mice model	NLRP3 inflammasome deficiency abolishes MPTP-induced microglial recruitment.	[99]
PD	Mice with microglial deletion of Atg5	NLRP3 inflammasome activation increases the expression of macrophage migration inhibitory factors and neuroinflammatory levels.	[100]
PD	*Atg5*^flox/flox^ mice intraperitoneally injected with MPTP to induce experimental PD model	NLRP3 inflammasome activation promotes neuroinflammation and dopaminergic neurodegeneration.	[101]
PD	MPTP-induced PD mice model	NLRP3 inflammasome activation promotes GSDMD cleavage and subsequent pyroptosis of microglia.	[102]
Spinal cord injury	Sprague–Dawley rat models with spinal cord injury	The ROS/TXNIP/NLRP3 signaling pathway aggravates neuroinflammation.	[107]
HD	R6/2 transgenic HD mice model	NF-κB/NLRP3 pathways contributes to neuroinflammation.	[108]
HD	R6/2 transgenic HD mice model	The NLRP3 inflammasome promotes pyroptosis of striatal neurons.	[109]
Atherogenesis	High-fat diet feeding	Hyperactivation of the NLRP3/caspase-1/IL-1β signaling pathway promote atherosclerosis.	[115]
Atherogenesis	Intraperitoneal injection of cholesterol crystals or high-cholesterol diet feeding	NLRP3 inflammasome activation and downstream cytokine release promote atherosclerosis.	[116]
Atherogenesis	High-fat diet feeding	Inhibition of thioredoxin-1/NLRP3 pathway has a protective effect on atherosclerosis.	[117]
Atherogenesis	Mice fed with high-fat diet or/and administrated with the water containing nicotine	ROS–NLRP3-mediated endothelial cell pyroptosis promotes atherosclerosis.	[118]
Dilated cardiomyopathy	Mice intraperitoneally injected with doxorubicin	NLRP3 inflammasome activation causes pyroptosis and myocardial dysfunction through caspase-1.	[121]
Heart failure	Mice undergo transverse aortic constriction surgery	Activation of NLRP3 inflammasome increases cardiac inflammation.	[122]
NASH	Mice fed with an atherogenic diet for 16 weeks, gavaged MCC950 until 24 weeks or mice fed a methionine/choline deficient diet, gavaged MCC950 for 6 weeks	Intracellular NLRP3 inflammasome activation enhances NASH inflammation.	[125]
NASH	Mice model of LPS/D-GalN-induced endotoxin acute hepatitis or fibrotic NASH resultant of long-term feeding with a high-fat, fructose, and cholesterol diet	Activation of NLRP3 inflammasome increases inflammation and promotes liver fibrosis development.	[132]
Liver cirrhosis	Patients	Activation of NLRP3 inflammasome increases plasma levels of IL-1β and IL-18 in patients with cirrhosis.	[134]
Liver cirrhosis	Mice intraperitoneally injected with CCL_4_ to induce liver cirrhosis	After NLRP3 inflammasome activation, IL-1β is secreted extracellularly by the GSDMD pore to exert an inflammatory effect.	[135]
Liver fibrosis	Mice intraperitoneally injected with CCL4 or thioacetamide to induce liver cirrhosis	NLRP3 inflammasome activation increases liver inflammation by releasing proinflammatory factors.	[139, 140]
Liver fibrosis	A hepatocyte-specific NLRP3 heterozygous gain of function mutant mouse strain	Hepatocyte NLRP3 inflammasome activation leads to hepatocyte pyroptosis and secretion of inflammasome complexes into the extracellular space.	[141]
IBD	Oral DSS administration	NLRP3 inflammasome plays a protective role in intestinal mucosa by reducing the production of proinflammatory factors.	[144]
IBD	Oral DSS administration	Defective NLRP3 inflammasome activation leads to loss of epithelial integrity and systemic dispersion of commensal bacteria.	[145]
IBD	*IL-10*^−/−^ mice	NLRP3 inflammasome is activated in colonic mucosa and aggravates colorectal inflammation.	[146]
IBD	Mice are given DSS orally or given a rectal administration of 2,4,6-trinitrobenzene sulfonic acid	Inhibition of NLRP3 inflammasome activation can alleviate the symptoms of colitis in mice.	[149, 150]
UC	Oxazolone is delivered intrarectally to mice	The NLRP3 inflammasome-derived IL-1β and IL-18 play a protective role against UC.	[151]
IBD	*Nlrp3*^R258W^ mutant mice	*NLRP3*^R258W^ enhances IL-1β secretion, which boosts local antimicrobial peptides to facilitate microbiota remodeling.	[152]
RA	Patients and healthy individuals	NLRP3 gene loci are associated with susceptibility to RA.	[155]
RA	Collagen‐induced arthritis mice model	Activation of NLRP3 inflammasome increases joint inflammation and bone destruction.	[156]
RA	Patients	Activation of NLRP3 inflammasome produces IL-1β in rheumatoid arthritis.	[157]
RA	Patients	CaSR-mediated NLRP3 inflammasome activation contributes to inflammatory arthritis.	[158]
Chronic active gouty arthritis	Patients with rilonacept treatment	Blocking the downstream cytokines of NLRP3 inflammasome can effectively reduce inflammation and pain in patients.	[161]
Gout	Intra-articular injection MSU in the knee of rats	BHB inhibits NLRP3 inflammasome to reduce gout.	[163]

### Autoinflammatory diseases

#### Cryopyrin-associated periodic syndrome

Cryopyrin-associated periodic syndrome (CAPS) is a group of rare genetic autoinflammatory diseases caused by mutations in the *NLRP3 *gene. CAPS can be classified into three subphenotypes according to the severity of the disease, including familial cold autoinflammatory syndrome (FCAS), Muckle–Wells syndrome (MWS), and neonatal multisystemic inflammatory syndrome (NOMID) (also known as chronic infantile neurocutaneous–articular syndrome, CINCA) [63]. In individuals with CAPS, gain-of-function mutations of the* NLRP3* gene result in the excessive activation of NLRP3 inflammasome that cause sustained and uncontrolled release of IL-1β. [64]. The pathogenesis of CAPS may be related to the dysfunction of some negative regulators of NLRP3. For example, caspase recruitment domain-containing protein 8 (CARD8) can bind to NLRP3 to prevent exogenous stimulation. However, CAPS-associated *NLRP3* mutations escape the control of CARD8 [65]. Furthermore, the cAMP–PKA directly inhibits the rapid activation of NLRP3 inflammasome, whereas CAPS-associated mutations disable this signaling pathway [66]. NLRP3 inflammasome activation-induced cytokine IL-1β is thought to be a key factor aggravating the inflammatory response [67] It is noteworthy that NLRP3 mutations appear to be specifically enhanced in neutrophils, with these cells being the primary source of IL-1β in severe CAPS, both in patients and in mouse models [68].

The current therapeutic approach for CAPS is to inhibit the downstream signaling pathway of IL-1, using the IL-1 receptor antagonist anakinra for clinical treatment [69, 70]. NLRP3 as an upstream component may be a potential new target. The current study found that the LRR sequence of NLRP3 plays an irreplaceable role in CAPS. Genes of the LRR sequence undergo alternative splicing after transcription, whose exons 4, 5, 7, and 9 determine whether NLRP3 inflammasome can be activated [71]. Importantly, this study shows that inhibition of LRR exons is sufficient to prevent the assembly of NLRP3 inflammasome, which could be a potential target for inhibitor development. Sensorineural hearing impairment is one of the common sufferings of CAPS patients. The NLRP3 inhibitor MCC950 significantly improved hearing impairment and systemic inflammation in CAPS mice [72]. In addition, the metalloproteinase inhibitor thiolutin prevented the deubiquitination of NLRP3 and thus the activation of multiple CAPS-associated NLRP3 inflammasome [27].

#### Familial Mediterranean fever

Familial Mediterranean fever (FMF) is a genetic disease caused by gene missense mutations. Mediterranean fever (*MEFV*) is the gene responsible for the pathogenesis of FMF and encodes the Pyrin protein. More than 50 mutations associated with FMF have been identified in MEFV [73, 74]. Pyrin can bind to ASC of multiple inflammasomes through its pyrin structural domain. In FMF, the pyrin inflammasome/caspase-1/GSDMD pathway causes the release of inflammatory cytokines and alarmins S100A8/A9 (hallmarks of FMF) extracellularly and exacerbates autoinflammation [75]. NLRP3 inflammasome trigger inflammation-related diseases mainly through the pro-inflammatory cytokine IL-1β. NLRP3 inflammasome appears to be more active in the population carrying *MEFV* mutations monocytes from FMF patients and healthy populations are treated with LPS, and monocytes from FMF patients secrete more IL-1β and positively correlate with the number and exocytosis of *MEFV* mutations. This IL-1β source is NLRP3-dependent, since silencing NLRP3 inhibits IL-1β secretion [76]. In addition, activation of the pyrin inflammasome in monocytes from FMF patients results in reduced expression of IL-1 receptor antagonists, making patients more sensitive to proinflammatory stimuli [77]. In contrast to the above studies, an animal study shows that the pathogenesis of FMF may not completely depend on the NLRP3 inflammasome, but rather be mediated by its downstream ASC or IL-1β agents[73]. The involvement of NLRP3 in the pathogenesis of FMF has not been fully elucidated. Furthermore, CAPS and FMF have similar pathogenesis and are both characterized by excessive IL-1 release, but their clinical manifestations are very different. Whether NLRP3 plays a key role in this is a potential research question.

### Neurodegenerative diseases

Neurodegenerative diseases are characterized by the progressive neuronal loss [78]. The NLRP3 inflammasome is involved in and drives the development of neurodegenerative diseases [79, 80].

#### Alzheimer’s disease

AD is the main cause of dementia and is characterized by cognitive loss and memory impairment [81]. It is evaluated that the number of dementia patients in the world has now exceeded 50 million, with the annual cost of more than one trillion US dollars [82, 83]. AD has emerged as a pressing public health concern [82]. One of the classic pathological features of AD is an intracellular hyperphosphorylated tau protein composition of neurofibrillary tangles (NFTs) [84]. It has been demonstrated that the formation of NFT caused by NLRP3 inflammasome exacerbates the development of AD [85] The deletion of the NLRP3 inflammasome can reduce the hyperphosphorylation and aggregation of tau protein by regulating phosphatase and tau protein kinase, which improves cognitive deficit in mice [86].

Neuroinflammation is an important cause of NFTs formation and accelerates the pathological progression of AD [87]. Neuroinflammation in AD is mainly driven by microglia, and a genome-wide association study found that microglia express proteins encoded by mutated genes associated with late-onset AD [88]. Tau released by neurons into the extracellular matrix can activate the NLRP3 inflammasome in microglia, which causes the extracellular release of downstream IL-1β and IL-18. Persistent signaling of the NLRP3 inflammasome induces microglial dysfunction, weakens the clearance capability of NFTs by microglia, and ultimately sets up a vicious proinflammatory cycle, accelerating neuronal cell death [86].

#### Parkinson’s disease

Parkinson’s disease (PD) is a neurological disorder with rare occurrence, and its main clinical manifestations are characterized by resting tremor, slow movement, stiffness, and loss of postural reflex [89]. PD is a complex disorder with many etiological factors and is related to genetics, environment, and their interaction [90, 91]. The classic pathological mechanisms of PD mainly include the damage and apoptosis of dopaminergic neurons in the substantia nigra compacta and the accumulation of α-synuclein (α-Syn) [92, 93].

In recent years, there has been a significant focus on neuroinflammation and immune dysfunction in PD [94]. Activation of NLRP3 inflammasome in peripheral blood mononuclear cells of PD patients results in increased plasma levels of α-syn and IL-1β, both of which aggravate the severity of dyskinesia in PD patients. Interestingly, plasma α-Syn levels in PD patients are positively correlated with proinflammatory factor IL-1β levels, suggesting that α-Syn released by degenerated neuron may act as an endogenous substance to activate NLRP3 inflammasome and induce a strong inflammatory response in PD [95]. The interaction between aggregated α-Syn and Toll-like receptor 2 may serve as the first initiating signal of NLRP3 inflammasome activation. Internalization of α-Syn by microglia leads to overproduction of mtDNA and mtROS, which act as the second signal to the NLRP3 inflammasome activation and ultimately induce neuroinflammation [96, 97]. Meanwhile, the NLRP3 inflammasome can release IL-1β through caspase-1 cleavage and exacerbate the inflammatory response by inducing pyroptosis. The entry of IL-1β into the substantia nigra site can promote the loss of dopaminergic neurons [98].

NLRP3 inflammasome activation in microglia also plays a pivotal role in PD. In the neurotoxin 1-methyl-4-phenyl-1,2,3,6-tetrahydropyridine (MPTP)-driven degeneration of dopaminergic neurons, the NLRP3 inflammasome activation in microglia significantly exacerbated dyskinesia and loss of dopaminergic neurons in mice [99]. In addition, microglial autophagy deficiency can result in the death of tyrosine hydrogenase neurons in the substantia nigra through NLRP3/PDE10A (phosphodiesterase 10A)–cyclic adenosine monophosphate (cAMP) signaling, the effects of which are reduced by NLRP3 inhibition [100]. The study by Qing et al. confirms this notion that autophagy deficiency in microglia can exacerbate MPTP-induced neurodegeneration in a mouse model by activating the NLRP3 inflammasome [101]

Drug therapy targeting the NLRP3 inflammasome improves the pathological progression of PD. Prussian blue nanozyme is a pyroptosis inhibitor, and can inhibit NLRP3 inflammasome activation by scavenging ROS as well as downregulate GSDMD autocleavage and pro-inflammatory factor production, attenuating MPTP neurotoxin induction neurodegeneration in mouse and cellular models of PD [102]. Additionally, dopamine inhibits canonical activation as well as noncanonical activation of the NLRP3 inflammasome in primary human microglia [103]. Andrographolide is a bicyclic diterpenoid lactone with immunomodulatory and antiinflammatory activities. Treatment of mice with andrographolide increases mitophagy, reduces activation of the NLRP3 inflammasome, and ultimately ameliorates the loss of dopaminergic neurons caused by the neurotoxin MPTP [104]. At present, the clinical drug therapy for PD still does not meet the ideal expectations. The animal experiments of antagonists targeting NLRP3 inflammasome and its related pathways have achieved favorable therapeutic effects. However, these drugs in the treatment of PD are intended for basic studies, and no clinical data have been evaluated.

#### Huntington’s disease

Huntington’s disease (HD) is an autosomal dominant neurodegenerative disease characterized by chorea, dystonia, motor incoordination, cognitive decline, and behavioral difficulties [105]. Galectin-3 (Gal-3) overexpression in microglia is one of the key causes of HD [106]. In a Sprague–Dawley rat model of spinal cord injury, Gal-3 aggravates neuroinflammation through the ROS/TXNIP/NLRP3 signaling pathway [107]. Similarly, Gal-3 expression is upregulated in the plasma of HD patients and mice. In vitro experiments further find that Gal-3 in the microglia of HD model produces IL-1β through the NLRP3 inflammasome-dependent pathway, aggravating brain inflammation [108].

Furthermore, studies in a transgenic R6/2 mouse model of HD (a model that exhibits a progressive neurological phenotype and mimics several features of human HD) find that NLRP3 and caspase-1 are highly elevated in the HD R6/2 mouse, which can further induce pyroptosis [109]. Notably, the NLRP3-specific inhibitor MCC950 treatment inhibits the activation of NLRP3 inflammasome, decreases IL-1β and ROS production, and ultimately improves motor dysfunction in mice [110]. Similarly, in another study, olaparib treatment downregulates the NLRP3 inflammasome and reduces caspase-1 cleavage in the R6/2 mouse model, ameliorating pyroptosis-induced missing neurons [111].

### Cardiovascular diseases

A study on the association between NLRP3-related gene mutations and cardiovascular diseases mortality reports that NLRP3 intronic variant rs10754555 is associated with inflammasome activation and systemic inflammation, and carriers of this gene have a higher risk of death [112]. Low-grade basal NLRP3 inflammasome activation promotes the development of various chronic cardiovascular diseases such as hypertension and atherosclerosis [7, 113]. Consequently, inhibiting the activation of the NLRP3 inflammasome is crucial for the treatment of cardiovascular disease. This section mainly focuses on the NLRP3 inflammasome in cardiovascular disease.

Atherosclerosis is a common cardiovascular disease characterized by lipid accumulation and persistent inflammation in large and medium arteries, which can eventually lead to various complications such as myocardial infarction and cerebral infarction [114]. Recently, several studies have manifested that the NLRP3 inflammasome serves as a potential therapeutic target for atherosclerosis by regulating the expression of pro-inflammatory factors [113, 115]. Multiple endogenous danger signals, such as cholesterol crystals [116], oxidized low-density lipoprotein, and some endogenous metabolites [117] activate the NLRP3 inflammasome, enhancing the expression of proinflammatory cytokines. Ultimately, these danger signals contribute to the development of atherosclerosis by enhancing oxidative stress and the inflammatory response. In addition, multiple cellular dysfunctions can lead to overactivation of the NLRP3 inflammasome to aggravate atherosclerosis. Mitochondrial uncoupling protein 1 (UCP1) is a critical factor in the thermogenesis and mitochondrial function of brown fat cells. UCP1 depletion leads to hyperactivation of the IL-1β and NLRP3 inflammasome by increasing mitochondrial membrane potential and mitochondrial superoxide, thereby exacerbating endothelial dysfunction, vascular inflammation, and atherosclerosis in obese mice [115]. In addition, smoking and other bad living habits are also contributing factors of atherosclerosis. Specifically, nicotine treatment of human aortic endothelial cells results in NLRP3 inflammasome activation and pyroptosis, the effects of which are reversed by NLRP3 silencing or ASC with small interfering RNA [118].

Heart failure (HF) is a syndrome in which cardiac output cannot be maintained under normal filling pressure, mainly caused by a variety of cardiac structural or functional abnormalities [119]. Notably, activation of the NLRP3 inflammasome produces inflammatory factors that recruits macrophages and T cells to trigger cardiomyocyte pyroptosis and cardiac inflammation, leading to fibrosis, adverse cardiac remodeling, and even heart failure [120]. Zeng et al. found that in dilated cardiomyopathy (DCM) patients and doxorubicin-induced DCM mice, the NLRP3 inflammasome triggers cardiomyocyte pyroptosis through the caspase-1 pathway [121]. However, empagliflozin reduce cardiac inflammation in rats with heart failure and exert a cardioprotective effect by inhibiting the NLRP3 inflammasome [122].

### Liver disease

#### Nonalcoholic steatohepatitis

Nonalcoholic fatty liver disease (NAFLD) is a clinicopathological syndrome that directly leads to cirrhosis and hepatocellular carcinoma [123, 124]. Nonalcoholic steatohepatitis (NASH) is one of the prime reasons of liver cirrhosis.

The *foz/foz* mouse is a model of over-nutrition with phenotypes of obesity and metabolic syndromes such as diabetes and hypercholesterolemia. *Foz/foz* mice fed a diet deficient in methionine and choline develop severe steatohepatitis and liver fibrosis. In the liver of *foz/foz* mice, cholesterol crystals promote the expression of NLRP3 and its downstream molecules caspase-1 and IL-1β. However, MCC950 treatment reduces the levels of these inflammatory factors and improves liver fibrosis [125]. Furthermore, in an obese mouse model fed a high-fat diet, the high-fat diet activates the hepatic NLRP3 inflammasome and enhances the expression of NLRP3, ASC, caspase-1, IL-6, and TNF-α, deteriorating NASH [126]. However, single-cell transcriptional profiling of the livers of *Nlrp3*^A350V^ mutant mice show that activated NLRP3 can cause NASH and liver fibrosis even in the absence of hepatic steatosis [127]. Therefore, steatosis may not be necessary for the NLRP3 inflammasome-induced fibrosis and NASH.

MicroRNAs have a variety of functions in regulating gene expression of inflammatory factors [128, 129]. The specifically expressed microRNA-223 (miR-223) is significantly downregulated in neutrophils of the liver of NASH patients and is considered to be a key negative regulator of NLRP3 expression [130]. In miR-223-deficient mouse liver macrophages, the mRNA expression of NLRP3 is increased, while significantly decreased after supplementation of mice with miR-223 3p (miR-223 endogenous analog [131]). Furthermore, in fibrotic NASH mice chronically fed a high-fat, fructose, and cholesterol diet, miR-223 3p reduces NLRP3 and IL-1β production, significantly ameliorating NASH [132].

#### Liver cirrhosis

Cirrhosis is a common chronic liver disease, and its pathological changes include diffuse degeneration and necrosis of hepatocytes, fibrous tissue hyperplasia, and nodular regeneration of hepatocytes. Cirrhosis leads to the replacement of healthy liver tissue by fibrotic tissue and regenerative nodules, ultimately causing the loss of normal liver function [133]. Previous studies have found higher NLRP3 and caspase-1 expression levels in liver of patients with cirrhosis [134]. The NLRP3 inflammasome is significantly activated in a CCL_4_-induced mouse model of cirrhosis, which aggravates hepatocyte death and cirrhosis through the NLRP3/caspase-1/GSDMD classical pyroptosis pathway [135].

Progressive liver fibrosis is a common pathological process of liver cirrhosis [136], and is characterized by excessive deposition of extracellular matrix (ECM). Hepatic stellate cell (HSC) activation plays a main role in the pathogenesis of liver fibrosis [137]. The activated NLRP3/caspase-1 signaling pathway forms mature IL-18 and IL-1β, which further promotes the transformation of HSCs into myofibroblasts and generates ECM [138]. However, MCC950 can not only inhibit the development of fibrosis, but also improved the function of fibrotic liver. Auvro et al. show that MCC950 also inhibits collagen expression and HSC activation in the mouse model of fibrotic NASH [125]. Similarly, in the CCL_4_-induced liver fibrosis model, alpinetin and auranofin also exert antiinflammatory and antioxidant effects by blocking the NLRP3 inflammasome [139, 140].

The release of inflammasome components caused by hepatocyte pyroptosis is another pathological mechanism of liver fibrosis. Susanne et al. found that activation of the NLRP3 inflammasome could lead to hepatocyte pyroptosis in mouse and human primary hepatocytes, whereas it can be inhibited by caspase-1 inhibitor by blocking the activation of GSDMD. Components of the inflammasome that are released extracellularly activate HSCs through endocytosis, increase the secretion of IL-1ß and the expression of α-SMA, and promote the development of fibrosis [141]. The study by Alexander et al. also proves this view [142]. *NLRP3* gene knockout in mice show liver cell pyroptosis, HSC activation, collagen deposition, severe neutrophil infiltration, and liver inflammation [142].

In addition, the abundance of *Stenotrophomonas maltophilia* is higher in the liver of patients with cirrhosis when compared with healthy controls. It is found that *Stenotrophomonas maltophilia* induces the formation of NLRP3 inflammasome complex by activating TLR4-mediated NF-κB signaling pathway, which drives cirrhosis to deteriorate to hepatocellular carcinoma in mice [143]. Together, these findings [142] suggest that the NLRP3 inflammasome activation plays an important role in the progression of liver fibrosis–cirrhosis–hepatocellular carcinoma.

### Inflammatory bowel disease

Inflammatory bowel disease (IBD), incorporating ulcerative colitis (UC) and Crohn’s disease (CD), is characterized by intestinal inflammatory lesions. The NLRP3 inflammasome has dual roles in IBD. On the one hand, activation of the inflammasome further enhances the inflammatory response, leading to aggravation of colon damage; on the other hand, under certain conditions, the inflammasome can inhibit the further development of inflammation and protects the colon from further damage [144, 145].

Dextran sulfate sodium (DSS) is a common method for constructing colitis models. In DSS mice, the degree of colitis in NLRP3 knockout mice is milder than that in wild mice, partly due to the reduced levels of proinflammatory cytokines [144]. In addition, in the colonic mucosa of UC patients, the NLRP3 inflammasome is significantly upregulated, and its activity gradually increased with the degree of disease progression [146]. Further studies found that DSS could enhance the NLRP3 inflammasome activation in macrophages by increasing the K^+^ efflux mediated by calcium-activated intermediate-conductance potassium ion channel (KCa3.1). In addition, genetic studies targeting CD found that NLRP3-related single-nucleotide site mutations are associated with susceptibility to CD [147]. CARD8, a negative regulator of the NLRP3 inflammasome, prevents NLRP3 from binding to ASC during inflammasome activation. Mutation of the gene encoding the CARD8 protein can lead to enhanced NLRP3 activity and increase downstream IL-1β secretion, resulting in the occurrence of CD [148]. Notably, some NLRP3 nature original inhibitors can improve colitis by restraining the activation of NLRP3. For example, cardamonin, a natural flavone isolated from *Alpinia katsumadai Hayata,* attenuates mice colitis by activating the AhR/Nrf2/NQO1 pathway and inhibiting the activation of the NLRP3 inflammasome [149]. Ginsenoside RK3, the main active ingredient of ginseng, can alleviate DSS-induced ulcerative colitis by inhibiting the expression of the NLRP3 inflammasome [150].

Paradoxically, the NLRP3 inflammasome also has a certain protective role in intestinal inflammation. Mice with *NLRP3*, *ASC*, or *caspase-1* gene knockout are more susceptible to DSS-induced colitis, which is a result of reduced IL-18, a downstream component of the NLRP3 inflammasome. Defective NLRP3 inflammasome activation results in loss of intestinal epithelial integrity, which in turn results in commensal bacterial overgrowth and bacteremia [145]. In addition, in the oxazolone-induced mouse UC model, NLRP3 knockout mice have more severe colitis, as indicated by increased Th2 cytokine expression and decreased production of mature IL-1β and IL-18. Exogenous administration of IL-1β reduces the expression of colonic Th2 cytokines IL-4 and IL-13 and improves colitis, while exogenous IL-18 also reduces the severity of colitis but does not affect the expression of Th2 cytokines [151]. Yao et al. found that *NLRP3*^R258W^ mutant mice have a gain-of-function mutation in the *NLRP3* gene coding region. Notably, the mice suffer from CAPS but do not develop autoinflammation because the *NLRP3*^R258W^ mutation-mediated remodeling of intestinal microbiota induces Treg cells to maintain homeostasis and improve intestinal anti-inflammatory ability [152].

### Arthritis

#### Rheumatoid arthritis

Rheumatoid arthritis (RA) is an inflammatory disease that primarily affects joints throughout the body, and can lead to cartilage and bone damage and even disability [153]. Many inflammatory factors such as TNF-α, IL-1β, and IL-6 are involved in the occurrence and development of inflammatory joint damage [154]. Genetic studies have shown that the C allele at rs4612666 and the G allele at rs10754558 of the* NLRP3 *gene coding locus can increase the risk of RA [155]. The NLRP3 inflammasome is mainly involved in RA progression through regulating downstream cytokines, of which inhibition of IL-1β is particularly significant for RA treatment [83]. The NLRP3 inflammasome in synovial fluid monocytes and macrophages is significantly activated in RA patients and in a mouse model of collagen-induced arthritis. MCC950 treatment significantly reduces IL-1β and improves joint inflammation and bone destruction in a mouse model [156]. In addition, RA disease itself may also trigger the activation of the NLRP3 inflammasome, forming a vicious circle and aggravating the development of the disease. However, anticitrullinated protein antibodies (ACPAs) (RA-specific autoantibodies) can induce the expression of NLRP3 and pro-IL-1β by activating the CD147/ITGB1/Akt/NF-κB signaling pathway. On the other hand, ACPAs can activate Pannexin channels to promote the release of ATP. Subsequently, the accumulated ATP binds to the P2X7 receptor leading to the activation of NLRP3 inflammasome [157]. Moreover, extracellular Ca^2+^ promotes calcium-sensing receptor signaling in RA, leading to the activation of the NLRP3 inflammasome and the release of IL-1β [158].

#### Gout

Gout is a recurrent inflammatory disease caused by the deposition of urate crystals in the synovium, synovial bursa, cartilage, and other tissues of joints. Activation of NLRP3 inflammasome and release of IL-1β by monosodium urate crystals are pivotal pathological factors in gout attacks [159]. Fabio et al. revealed that monosodium urate (MSU) is involved in the activation of the NLRP3 inflammasome and promotes the production of IL-1β and IL-18 [160]. Some human experiments have shown that IL-1β inhibitors rilonacept, canakinumab, and anakinra are effective in the treatment of acute and chronic gout patients [161, 162]. In addition, a ketogenic diet (KD) can inhibit NLRP3 inflammasome activation by increasing the level of beta-hydroxybutyrate in neutrophils, thereby blocking IL-1β production and alleviating gout [163]. In conclusion, the NLRP3 inflammasome is expected to be a future therapeutic target for gout.

## Summary and perspectives

The innate immune system provides the host with the first line of defense against pathogens, but continued activation of this system can lead to several diseases. The NLRP3 inflammasome is a key component of the innate immune system and plays an important role in inflammation-related diseases such as atherosclerosis, AD, and IBD through promoting the release of IL-1β and IL-18. This review mainly summarized the various activating factors of NLRP3 inflammasome (Fig. 1) and emphasizes its regulatory mechanism in the above-mentioned diseases, providing new ideas for targeting NLRP3 inflammasome to treat inflammation-related diseases. The activation mechanism of NLRP3 inflammasome mainly includes K^+^ efflux, mitochondrial dysfunction, and lysosome rupture. However, most of the current understanding of the pathogenic mechanism of NLRP3 inflammasome comes from animal experiments, and how it is activated and regulated in humans has not been fully elucidated. Therefore, the study of NLRP3 inflammasome compositions, downstream pathways, tandem pathways, and the development of related inhibitors have broad prospects in the treatment of a wide range of inflammation-related diseases.

A variety of NLRP3 inhibitors have been found to target the NLRP3 inflammasome, some of which directly target the NLRP3 protein, while others target other components and downstream products of NLRP3 inflammasome. For example, the monoclonal anti-IL-1β antibody canakinumab can rapidly relieve the symptoms of CAPS patients and has a high safety profile [164, 165]. In addition, NLRP3-specific small-molecule inhibitor MCC950 has shown promising efficacy in various animal models of different diseases, such as autoinflammatory diseases, cardiovascular diseases, cancer, neurological diseases, and diabetes[166–169]. MCC950 can inhibit ATP hydrolysis and NLRP3 inflammasome formation by interacting with the Walker B motif in the NACHT domain of NLRP3 protein [170–172]. Another endogenous small molecule inhibitor BHB reduces K^+^ efflux and ASC speck-like aggregates formation to inhibit NLRP3 inflammasome activation, decreasing the levels of IL-1β and IL-18 produced by NLRP3 inflammasome in human monocytes [173]. BHB has been confirmed to play an anti-inflammatory role in the animal models of AD, gout, and acute kidney injury, and is expected to become a potential clinical drug [163, 174, 175]. However, the clinical application of NLRP3 inhibitors also has a lot of limitations. Previous studies have shown that the pharmacokinetics and toxicokinetics of MCC950 limit its clinical application [176]. Although BHB can prevent chronic progressive diseases such as diabetes and AD, BHB has a poor therapeutic effect in the face of acute inflammatory diseases. Improving the defects of existing NLRP3 inflammasome inhibitors, such as reducing the hepatotoxicity of MCC950, or developing new NLRP3 inflammasome targeting inhibitors is an urgent problem to be solved, which may provide new idea for solving clinically related diseases.

In addition, noncoding RNAs, such as microRNAs, also provide new possibilities for inhibiting the NLRP3 inflammasome [131]. LncRNA 4344 directly reduces the expression of NLRP3 and its downstream genes by targeting miR-138-5p, and improves cognitive behavior, neuroinflammation, and apoptosis in rats with LPS-induced cognitive impairment [177]. Long noncoding RNA XLOC_000647 can inhibit NLRP3 promoter activity and reduce NLRP3 expression at the transcriptional level in mice with pancreatic cancer, subsequently retarding cancer cells proliferation, invasion, and the epithelial–mesenchymal transition of in vitro [178]. Noncoding RNAs can regulate the expression and function of NLRP3 inflammasomes more finely at the gene level. Therefore, an in-depth understanding of its mechanism may help develop more precise and effective targets for NLRP3 inhibition.

Notably, targeting NEK7 provides a new direction for the development of NLRP3 inhibitors. NEK7 is a serine/threonine protein kinase that helps spindle formation and drives mitosis [179]. The formation of the NLRP3–NEK7 complex is one of the critical steps in the activation of the NLRP3 inflammasome [36, 37]. Previous studies have shown that artemisinin improves joint swelling in arthritic mice through inhibiting the NLRP3–NEK7 interaction and attenuating LPS- and MSU-induced K^+^ efflux in macrophages [180]. Novelly, RRx-001, a well-tolerated anticancer drug, can also block NLRP3–NEK7 interaction, alleviating colitis induced by DSS, systemic inflammation induced by LPS, and experimental autoimmune encephalomyelitis in mice [181]. Overall, most of the studies on NLRP3 targeting inhibitors are still at the stage of animal experiments. There are some NLRP3 inhibitors have entered early clinical trials, but the best clinical indications for NLRP3 inflammasome blockade remain to be determined [182]. The broad role of NLRP3 in disease poses a great challenge in selecting the indication for the best efficacy.

Interestingly, the NLRP3 inflammasome has two sides in inflammation-related diseases. The NLRP3 inflammasome exhibits deleterious effects in most diseases, including inflammatory diseases, metabolic diseases, and most tumors [183]. However, in some cancers, infectious diseases, and IBD, the NLRP3 inflammasome has different roles [151, 183]. For example, the NLRP3/IL-1 signaling axis induces the expansion of bone marrow-derived suppressor cells in melanoma, which reduces the activity of natural killer cell and CD8^+^ T cell activity and accelerates tumor growth [184]. However, lack of the NLRP3 inflammasome results in impaired IL-18 signaling, which in turn leads to liver metastasis of colorectal cancer in mice with colorectal cancer [185]. However, the reasons for the above differences are still unknown, which may be related to the involvement of NLRP3 inflammasome in different signaling pathways. How to precisely regulate the activation and function of the NLRP3 inflammasome so that it can play a more protective role in different organs and diseases may be a new direction for future research.

In conclusion, the research on NLRP3 and inflammation-related diseases has made some progress, but there are still many challenges, such as the precise regulation of NLRP3 inflammasome activation and function, and the development and clinical application of NLRP3 targeted inhibitors. However, the complicated interaction of different molecular pathways involved in NLRP3 activation increases the difficulty of targeted therapy. In the meantime, elucidating the mechanisms by which NLRP3 inflammasome plays dual roles in some diseases, such as IBD and colorectal cancers, will promote the research of inflammation-related diseases in the future. Therefore, based on the previous researches, a lot of basic and clinical researches are still needed to achieve targeting the NLRP3 inflammasome in the treatment of inflammation-related diseases.

## Data Availability

Not applicable.

## Abbreviations

Aβ: Amyloid β
ACPAs: Anticitrullinated protein antibodies
AD: Alzheimer’s disease
ASC: Apoptosis-associated speck-like protein
α-Syn: α-Synuclein
cAMP: Cyclic adenosine monophosphate
CAPS: Cryopyrin-associated periodic syndrome
CARD: Caspase recruitment domain
CD: Crohn’s disease
CINCA: Chronic infantile neurologic, cutaneous, and articular
CMPK2: Cytidine monophosphate kinase 2
CNKI: China National Knowledge Infrastructure
DAMPs: Damage-associated molecular patterns
DCM: Dilated cardiomyopathy
DSS: Dextran sulfate sodium
ECM: Extracellular matrix
FADD: Fas-associated with death domain protein
FCAS: Familial cold autoinflammatory syndrome
FMF: Familial Mediterranean fever
Gal-3: Galectin-3
GSDMD: Gasdermin D
HD: Huntington’s disease
HF: Heart failure
HSC: Hepatic stellate cell
IBD: Inflammatory bowel disease
IL-1β: Interleukin-1β
IKK: Inhibitory kappa B kinase
KD: Ketogenic diet
K2P: Two-pore domain potassium
LPS: Lipopolysaccharide
LRR: Leucine-rich repeat
NACTH: Nucleoside triphosphatase domain
NAFLD: Nonalcoholic fatty liver disease
NASH: Nonalcoholic steatohepatitis
NEK7: NIMA (never in mitosis gene A)-related kinase 7
NFTs: Neurofibrillary tangles
NLR: NOD-like receptors
NLRP3: NOD-like receptor protein 3
NOMID: Neonatal-onset multisystem inflammatory disorder
*MEFV*: Mediterranean fever
miR: MicroRNA
MPTP: 1-Methyl-4-phenyl-1,2,3,6-tetrahydropyridine
MSU: Monosodium urate
MWS: Muckle–Wells syndrome
mtDNA: Mitochondrial DNA
mtROS: Mitochondria active oxygen species
OX-mtDNA: Oxidized mitochondrial DNA
PAMPs: Pathogen-associated molecular patterns
PD: Parkinson’s disease
PDE10A: Phosphodiesterase 10A
PRRs: Pattern recognition receptors
PTMs: Posttranslational modifications
PYD: Pyrimidine domain
RA: Rheumatoid arthritis
ROS: Reactive oxygen species
TGN: Trans-Golgi network
TLR4: Toll-like receptor 4
TNF-α: Tumor necrosis factor-α
TWIK2: Two-pore domain weak inwardly rectifying K^+^ channel 2
TXNIP: Thioredoxin-interacting protein
UC: Ulcerative colitis
UCP1: Uncoupling protein 1
